# Randomised controlled trial of thermostatic mixer valves in reducing bath hot tap water temperature in families with young children in social housing: A protocol

**DOI:** 10.1186/1745-6215-9-14

**Published:** 2008-03-19

**Authors:** Denise Kendrick, Jane Stewart, Carol Coupland, Michael Hayes, Nick Hopkins, Debbie McCabe, Robert Murphy, George O'Donnell, Ceri Phillips, David Radford, Jackie Ryan, Sherie Smith, Lindsay Groom, Elizabeth Towner

**Affiliations:** 1 Division of Primary Care, Floor 13, Tower Building, University of Nottingham, University Park, Nottingham, NG7 2RD, UK; 2 Nottingham Primary Care Research Partnership, Nottinghamshire County Teaching PCT, Hucknall Health Centre, Curtis Street, Nottingham, NG15 7JE, UK; 3 Child Accident Prevention Trust. 4th Floor, Cloister Court, 22–26 Farringdon Lane, London, EC1R 3AJ, UK; 4 Glasgow Housing Association, Granite House, 177 Trongate, Glasgow, G1 5HF, UK; 5 East End Community Homes, 1221 Gallowgate, Unit 60, The Forge Shopping Centre, Glasgow, G31 4EB, UK; 6 City Building (Glasgow) Limited Liability Partnership (LLP), Milton Service Centre, 450 Ashgill Road, Glasgow, G22 6HJ, UK; 7 Institute for Health Research, Swansea University, Singleton Park, Swansea, SA2 8PP, UK; 8 NHS Greater Glasgow and Clyde NHS, Dalian House, 350 St. Vincent Street, Glasgow, G3 8YZ, UK; 9 East End Child Safety Project, 503 London Road, Glasgow, G40 1NQ, UK; 10 Centre for Child and Adolescent Health, University of the West of England, Hampton House, Cotham Hill, Bristol, BS6 6JS, UK

## Abstract

**Background:**

Each year in the UK 2000 children attend emergency departments and 500 are admitted to hospital following a bath water scald. The long term effects can include disability, disfigurement or psychological harm and repeated skin grafts may be required as the child grows. The costs of treating a severe scald are estimated at 250,000 GBP. Children living in the most deprived wards are at greatest risk of thermal injuries; hospital admission rates are three times that for children living in the least deprived wards.

Domestic hot water, which is usually stored at around 60 degrees Celsius, can result in a second-degree burn after 3 seconds and a third-degree burn after 5 seconds. Educational strategies to encourage testing of tap water temperature and reduction of hot water thermostat settings have largely proved unsuccessful. Legislation in the USA mandating pre-setting hot water heater thermostats at 49 degrees Celsius was effective in reducing scald injuries, suggesting passive measures may have a greater impact. Thermostatic mixer valves (TMVs), recently developed for the domestic market, fitted across the hot and cold water supply pipes of the bath, allow delivery of water set at a fixed temperature from the hot bath tap. These valves therefore offer the potential to reduce scald injuries.

**Design/Methods:**

A pragmatic, randomised controlled trial to assess the effectiveness of TMVs in reducing bath hot tap water temperatures in the homes of families with young children in rented social housing. Two parallel arms include an intervention group and a control group where the intervention will be deferred.

The intervention will consist of fitting a TMV (set at 44 degrees Celsius) by a qualified plumber and provision of educational materials. The control arm will not receive a TMV or the educational materials for the study duration but will be offered the intervention after collection of follow-up data 12 months post randomisation.

The primary outcome measure will be the bath hot tap water temperature. Fifteen families per arm are required to detect a reduction in the mean bath hot tap water temperature from 60.4 degrees Celsius (SD 9.1) in the control group to 46 degrees Celsius in the intervention group, with 90% power and a 5% significance level (2 sided). Secondary outcome measures including acceptability will require a sample size of 120 participants.

**Discussion:**

Whilst TMVs have the potential to reduce scald injuries, to date there have been no randomised controlled trials assessing their effectiveness, acceptability and cost effectiveness.

**Trial Registration:**

ISRCTN21179067

## Background

Each year in the UK approximately 2000 children attend emergency departments and 500 are admitted to hospital following a bath water scald. Most (86%) admissions occur in children under 5 years of age and 67% of these involve a prolonged inpatient stay (5 days or more) or transfer to a specialist hospital or burns unit [1]. There has been no discernible downward trend in attendances [1] or admissions for bath water scalds over recent years [2]. The long-term effects can include disability, disfigurement or psychological harm and repeated skin grafts may be required as the child grows. The cost of treating a severe scald has been estimated at £250,000 [1]. Those at greatest risk of burn and scald injuries are those living in the most deprived wards, where the hospital admission rate is three times that for children living in the least deprived wards [3].

Bath hot tap water scalds most commonly occur amongst pre-school children, either when a child falls or climbs unsupervised into the water. More rarely a child or his/her sibling turns on the hot tap, or parents or siblings put a young child into bath water that is too hot [1,4]. Younger children are more likely to suffer more severe scalds, with a greater body surface area being involved than in scalds in older children [1,5].

Water at 60 degrees Celsius can result in a second-degree burn after 3 seconds and a third-degree burn after 5 seconds in adults [1], with even shorter exposures required to produce burns in children [6]. The Child Accident Prevention Trust therefore recommends a bath hot tap water temperature no higher than 46 degrees Celsius [7]. However, boiler and immersion heater thermostats are frequently set at 60 degrees Celsius or above [8,9]. A UK study of over 200 electric immersion heaters found 71% were set at temperatures above 65 degrees Celsius [10] and a recent survey of 165 homes in Wakefield, UK, found a mean bath hot tap water temperature of 60.4 degrees Celsius [11]. Infants' and toddlers' baths are typically in the range 32 to 41 degrees Celsius and adults' baths in the range 34 to 45 degrees Celsius [8]. Hence current tap water temperatures in many homes are high enough to cause severe burns and are much higher than the temperatures that families usually bathe at.

Scald prevention interventions aimed at encouraging families to test their hot water temperature have had only limited success. Fewer than half the families who received counselling by paediatricians and a free thermometer, tested their water temperature [12]. A mass media campaign which provided 140,000 free thermometers did not result in an increase in hot tap water temperature testing, and only half of those that tested their water and found it to be above 54 degrees Celsius lowered the water heater thermostat temperature [13]. Interventions aimed at encouraging families to reduce water heater thermostat settings in Canada and Australia have also had limited success; either because the majority did not reduce the setting [14] or reduced it insufficiently to reach the recommended safe setting [15].

Interventions not requiring active participation by families have been more successful in reducing tap water temperatures. Legislation in the USA in 1983 requiring new water heaters to be pre-set at 49 degrees Celsius was associated with a reduction in the percentage of homes with tap water temperatures above 54 degrees Celsius from 80% to 23% over the five year period post legislation [16]. Hospital admission rates, the total body area burned, the proportion requiring grafting and the proportion scarred were all lower in the period 1979 to 1988 than in the 1970s [16].

Thermostatic mixer valves do not rely on active participation by families. They restrict hot bath tap water temperatures but do not reduce the temperature of stored hot water or interfere with heating systems, and allow a supply of hotter water to different sites within the home. They are fitted across the hot and cold water supply pipes to the bath, allowing the hot bath tap water to be set at a fixed temperature, in spite of pressure and temperature fluctuations in the water supply system. A pilot project in the USA fitted 20 anti-scald devices in the homes of families with pre-school children in 1993. These devices were effective in reducing tap water temperature, but 19 of the 20 needed to be removed in the first 9 months after fitting due to sediment build up [17]. A recent small uncontrolled study in California found a lower hospital admission rate for childhood scalds in areas which received a scald-prevention program which included fitting anti-scald devices in 37 homes, but only 60% of valves remained fitted and functional 6 to 12 months after fitting [18]. An evaluation of the 'Hot Water Burns Like Fire' campaign in Queensland, which included legislation mandating fitting hot water tempering valves in new homes, those undergoing major renovation or having a new hot water system, found a significantly higher hot water temperature and scald injury rate post-legislation than pre-legislation [19]. To date there have been no randomised controlled trials assessing the effectiveness, acceptability and cost-effectiveness of thermostatic mixing valves.

Funding is provided for social housing in England by the Housing Corporation. Recent amendments to its Scheme Development Standards for housing projects receiving Social Housing Grant require TMVs to be fitted to all hot water taps in properties for older people and to bath taps in all other properties [20]. In Scotland, changes were made to the Scottish Building Standards in 2006, requiring TMVs to be fitted in homes being built or undergoing major refurbishment [21]. However, there is still no requirement for TMVs to be fitted in general housing in England and Wales, and demonstrating the effectiveness and cost-effectiveness of TMVs will be important in driving TMV policy development.

### Objectives

The objectives of this research are to:

1. assess the effectiveness and cost effectiveness of TMVs in reducing bath hot tap water temperature in the homes of families with pre-school children living in rented social housing

2. assess the acceptability of TMVs to families with pre-school children living in rented social housing

3. assess the impact on bath time safety practices (i.e. adverse and potentially adverse effects from fitting TMVs, such as use of less safe methods of obtaining hotter bath water, reduced supervision or omitting to check water temperature on the assumption that it is safe)

### Design

Pragmatic parallel arm randomised controlled trial to assess the effect of TMVs in reducing bath hot tap water temperatures in the homes of families with young children in rented social housing.

### Participants

Families with pre-school children living in Glasgow Housing Association (GHA) housing. Families will be excluded if they are not GHA tenants, if their bathroom pipe work is found to be unsuitable for fitting a TMV or if they are already participating in on-going tap water scald prevention studies.

A range of recruitment strategies will be used. These will include:

(a) written invitation from GHA to (i) GHA tenants on the East End Child Safety Project (EECSP) Database, which is a database of families who have taken part in a previous child safety project and (ii) to tenants aged 18 to 40 years identified from the GHA tenant database.

(b) face-face contact with (i) local housing organisations (LHOs) who will provide information about the study to families due to have kitchen or bathroom refurbishments or other repairs which necessitate the LHO to visit the family's home and to families at open days and other events, and (ii) local projects or organisations such as the EECSP and the Parents and Children Together (PACT) team will provide information about the study to GHA tenants they meet during project activities

(c) articles describing the study will be placed in LHO newsletters and local newspapers and GHA tenants who would like to know more about the study will be asked to contact the research team.

Families expressing interest in participating will be contacted by the research team to discuss the study, answer any questions and assess eligibility. Those agreeing to participate will be asked to complete a baseline questionnaire, which families can choose to complete by telephone or by post.

### Interventions

Participating families will be randomised to the intervention or control arm. The two arms of the trial are:

Intervention arm, where families will be offered:

• An educational leaflet devised by the research team sent to the participant prior to installation of TMV.

• A TMV valve fitted to the hot and cold bath water supply by a qualified plumber from City Building (Glasgow) Limited Liability Partnership. The maximum temperature at which this will be set will be 44 degrees Celsius (the valves have a +/- 2 degrees Celsius tolerance; hence the maximum water temperature should not exceed 46 degrees Celsius).

• An educational hanger on how to use TMV to be attached to the tap by the plumber at installation.

Control arm, where families will be offered the intervention *after *collection of follow-up data.

### Outcome measures

#### Definition of primary and secondary outcome measures

The primary outcome measure will be the bath hot tap water temperature at 3 and 12 months post installation of TMV (intervention group) or post randomisation (control group).

Secondary outcome measures will include:

a) Intervention arm only:

• TMV problems (failures, replacements, repairs or removals and reasons why TMVs are repaired, replaced or removed).

• Adjustment of temperature setting of TMVs by families.

• Satisfaction with TMVs.

• Cost of fitting TMVs and of any TMV problems.

b) Both intervention and control arms:

• A bath hot tap water temperature = 46 degrees Celsius.

• Satisfaction with bath hot tap water.

• Bath time safety practices (e.g. bath time supervision, use of other methods to increase bath water temperature, checking bath water temperature).

#### Ascertainment of outcomes

##### Baseline data collection

The baseline questionnaire will include questions on socio-demographic and economic characteristics, bath time safety practices, bath time supervision and satisfaction with hot water supply. Families completing the baseline questionnaire will be offered an incentive (free bath mat or first aid kit) as incentives have been shown to increase response rates [22]. The design of the baseline questionnaire will be informed by individual face to face interviews with a sample of approximately 15 families residing in rented social housing. A purposive sample of families will be obtained to include families with differing maternal educational levels, different family composition, including children of differing ages, families with differing ethnic origins and differing levels of disadvantage. Interviews will take place in the families' homes and will explore issues surrounding bath safety and bath time supervision.

The first 50% of participants recruited to each arm will have their bath hot tap water temperature measured at recruitment to the trial. Water temperatures will be determined by direct measurement by a qualified plumber from City Building (Glasgow) Limited Liability Partnership, using a Type K thermocouple thermometer and rounded immersion probe. The temperature of the hot water supply will also be measured at an outlet other than a bath and without a TMV e.g. wash basin. This will enable detection of other methods to reduce hot water temperature such as reducing water heater thermostat settings.

#### Follow-up data collection

##### Primary outcome measure

A random sample of 15 families in each arm, who did not have their tap water temperature measured at baseline, will be chosen to have their tap water temperature measured at 3 months post fitting of TMVs and a separate random sample of 15 families in each arm will be selected to have their tap water temperature measured at 12 months post fitting of TMVs. Families will be offered a £10 voucher for use in local stores to increase response rates for water temperature measurements [22]. Measurements will be made in the same way as at baseline.

##### Secondary outcome measures

Secondary outcomes will be measured at 12 months post installation of TMVs (intervention arm) and at 12 months post randomisation (control arm). Data will be collected by questionnaire, which families can choose to complete by telephone or by post. The follow-up questionnaire will include questions on bath time safety practices, bath time supervision and satisfaction with hot water supply. Intervention arm families will also be asked questions about satisfaction with the TMV and the fitting process, attempts to adjust the TMV temperature setting, TMV problems, occupational details and work time lost as a result of TMV fitting or problems. TMV problems will also be ascertained from repair requests or complaints made to GHA by families. Families who complete the follow-up questionnaire will be offered a £5 gift voucher for use in local stores to increase response rates [22].

The design of the follow-up questionnaire will be informed by interviews with a sample of approximately 15 families in rented social housing who have had TMV valves fitted. A purposive sample of families will be obtained, as described above, but also, if possible, to include families with differing sites of installation of TMVs, and those who have experienced TMV failures. Interviews will be used to explore families' attitudes towards TMVs, experiences of using TMVs, attempts at altering temperature settings on TMVs and any changes in safety behaviour or bath time supervision following the fitting of TMVs. They will also be used to inform the development of questions on satisfaction with TMVs, bath time safety and supervision.

The cost-effectiveness analysis will be conducted from the perspective of public services and families. Costs associated with purchasing, fitting, maintaining, repairing and replacing TMVs will be estimated by GHA based on the unit cost of a TMV and associated pipe work plus the time required for plumbers to remove and re-fit the bath panel (or bath if necessary), to install the TMV and to test the bath hot tap water temperature before and after fitting the TMV. Participants' occupation, length of time off work and other costs incurred for TMV fitting or for repair or replacement in the event of TMV problems will be ascertained on the follow-up questionnaire and work time loss will be costed using relevant published wage rates. In addition, the costs associated with provision of the educational leaflet will be included in the cost profile of the intervention.

### Sample size

Ten families per arm are required to detect a reduction in the mean bath hot tap water temperature from 60.4 degrees Celsius (SD 9.1)[11] in the control group to 46 degrees Celsius in the intervention group, with 90% power and a 5% significance level (2 sided). Allowing 33% for losses to follow-up, 15 families in each arm will be randomly selected for tap water temperature testing at 3 and 12 months post valve installation (intervention arm) or randomisation (control arm). A different 15 families in each arm will be tested at 3 and 12 months.

For the secondary outcome measures, 50 families in each arm would provide:

• 80% power at the 5% significance level (1 sided) to test the hypothesis that the proportion of TMVs removed, disabled or whose temperature setting was adjusted did not exceed 6.5% if the true proportion of valves removed, disabled or adjusted was 1%.

• 80% power at the 5% significance level (2 sided) to detect a difference in the proportion of families agreeing that their bath hot tap water temperature was sufficiently hot from 90% in the control arm to 67% in the intervention arm.

To allow for losses to follow-up, a total of 120 participants will be recruited.

### Randomisation

After giving informed consent, families will be randomised to treatment arms. The randomisation schedule will be generated by the trial statistician using a computerised random-number generator in Stata [23], using a permuted block design with a random block size. Equal numbers will be allocated to the two groups. No stratification will be used. Group allocations will be placed in sequentially numbered, opaque, sealed envelopes. These will be opened sequentially by a researcher independent of the research team after participants have consented. The independent researcher will inform the study research fellow of the allocation who will inform GHA of the allocation. The research fellow will write to each family informing them of the allocation and provide details of how they will be contacted by GHA to organise water temperature measurement and TMV fitting.

### Blinding

It is not possible to blind participants, plumbers or researchers to treatment group allocation. Data will be analysed blind to treatment group allocation.

### Withdrawals

Participants will be free to withdraw from the trial at any stage. Those who move from the property before follow-up will be withdrawn from the trial.

### Analysis

The analysis will conform to a pre-specified analysis plan. Bath hot tap water temperatures will be described using the mean and standard deviation, or median and interquartile range, depending on the distribution of temperatures. The primary analysis will compare temperatures between treatment arms using an unpaired t-test to estimate the difference between the means and its 95% CI, assuming assumptions for undertaking these analyses are met. If assumptions are not met and no suitable transformation can be found, temperatures will be compared between treatment arms using the Mann-Whitney U test.

An intention to treat analysis will be undertaken replacing missing 3 and 12 month water temperatures (excluded participants and those where access could not be gained to homes for temperature measurement), with baseline temperatures, or where baseline temperatures are unavailable they will be replaced by the mean or median temperature for that treatment arm, dependent on the distribution of the temperatures.

The proportion of families with a bath hot water temperature ≤ 46 degrees Celsius will be compared between treatment arms using χ^2 ^tests and logistic regression to estimate the odds ratio and its 95% CI. Outcomes measured on an ordinal scale will be compared between treatment arms using χ^2 ^tests for trend, or will be dichotomised and analysed as binary variables depending on the distribution of responses.

Sensitivity analyses will be undertaken to examine the effect of adjusting for baseline temperature. Where baseline temperatures are unavailable they will be replaced by the mean or median temperature for that treatment arm, dependent on the distribution of the temperatures. These analyses will be undertaken using linear regression or a Mann-Whitney U test based on change scores.

The proportion of families in the intervention arm who have TMVs removed, replaced or repaired and who adjust the temperature setting of the TMV will be described using proportions and 95% CI.

#### Health economic analysis

Cost-effectiveness will be determined by comparing cost differences between intervention and control groups with differences in the number of families in each group with a maximum bath water temperature of 46 degrees Celsius. The reduction in scald-risk resulting from reduced bath water temperature will also be assessed and the cost per percentage reduction in risk estimated. A series of sensitivity analyses will be applied to the baseline findings to assess the robustness of the cost-effectiveness ratios generated.

#### Qualitative Analysis

Framework analysis [24] will be used to analyse interview data. This is a structured method of qualitative data analysis, where a priori themes are identified, but emergent themes can also be identified and incorporated into the analytic framework.

### Risks

We do not anticipate that participants will be exposed to any excess risk through participating in the trial. However, it is plausible that the installation of a TMV could impact on bath time safety practices, which may affect the risk of other bath time injuries. In attempt to minimise this risk we will reinforce bath time safety messages using the educational leaflet (prior to TMV installation) and the bath tap hanger, (at the time of installation). Bath time safety practices are also being measured as secondary outcomes.

### Ethical and organisational review

The trial protocol was reviewed by Nottingham Research Ethics Committee 1. As the trial does not involve NHS staff or patients and hence does not fall within the remit for NHS ethics committee approval, the committee were able to provide a review, but not approval. The trial received NHS organisational approval from Nottinghamshire County Primary Care Trust (PCT), (formerly Broxtowe and Hucknall PCT) as some research staff working on the trial were employed by the PCT.

## Discussion

This trial will enable an evaluation of the effectiveness and cost-effectiveness of TMVs fitted in the homes of families with young children in social housing. It will also assess families' satisfaction with the TMVs and the fitting process and potential unintended adverse events.

We considered that a randomised controlled trial was necessary to evaluate the effectiveness of TMVs as a non-randomised comparison may result in systematic differences between the intervention and control arms in interest and motivation to reduce bath hot tap water temperature. Such differences may impact on satisfaction with water temperature, which is likely to influence uptake of TMVs, hence an unbiased assessment of this outcome is crucial.

The trial will not be adequately powered to detect a reduction in the incidence of bath tap water scalds, and a very much larger trial would be required to do this. However, if TMVs are effective at maintaining a bath hot tap water temperature at 46 degrees Celsius or below, water at this temperature would take more than 9 minutes to cause a second degree burn [8]. Such a reduction is likely to virtually eliminate most tap water scalds [8]. Hence bath hot tap water temperature is likely to be a good proxy measure for bath tap water scalds.

As there is a steep social gradient in thermal injuries we considered it important to test the effectiveness of TMVs in families in rented social housing. However, this is likely to be a hard to reach group. Illiteracy in Scotland is strongly associated with low income, belonging to a manual social class and living in a deprived area [25], all of which characterise this study population. Furthermore parents living in poverty may fail to engage with services because they are viewed with suspicion [26]. In addition families with young children and those in social housing are more mobile than other families [27], and as TMVs are fitted to the home, only participants still resident in their homes can be followed up, so withdrawals may be high. Consequently the trial is likely to experience challenges in recruitment, maintenance of participants, access to homes to fit TMVs and measure water temperatures and in follow-up of participants. We are using a range of recruitment methods to try and address these issues, including face-to-face methods with known or trusted sources (e.g. the East End Child Safety Project and the PACT team). We are also using incentives to increase response rates for baseline and follow-up questionnaires and to encourage water temperature measurements, and providing participants with a choice of how questionnaires are completed to overcome literacy problems. Participants are being offered several appointments for TMV fittings and for water temperature measurements, with telephone and postcard reminders of appointments.

This trial will be the first randomised controlled trial to provide data on the real world effectiveness of thermostatic mixing valves in reducing hot bath water temperature in a disadvantaged population. Such information will be useful for policy makers, social housing providers, families and injury prevention practitioners.

## Competing interests

The author(s) declare that they have no competing interests.

## Authors' contributions

DK had the original idea for the study, obtained funding and drafted the protocol. JS, LG, CC, MH, ET and CP obtained Department of Health funding and helped with drafting of the protocol. NH obtained funding from Glasgow Housing Association and helped with drafting the protocol. DR obtained funding from Greater Glasgow and Clyde NHS and helped with drafting the protocol. DM, RM, GO, JR and SS helped with drafting the protocol. All authors read and approved the final manuscript.

## References

[B1] Department of Trade and Industry Government (1999). Consumer safety research: Burns and scalds accidents in the home.

[B2] Eadie PA, Williams R, Dickson WA (1995). Thirty-five years of paediatric scalds: are lessons being learned?. British Journal of Plastic Surgery.

[B3] Hippisley-Cox J, Groom L, Kendrick D, Coupland C, Webber E, Savelyich B (2002). Cross sectional survey of socioeconomic variations in severity and mechanism of childhood injuries in Trent 1992-7. British Medical Journal.

[B4] Yeoh C, Nixon JW, Dickson W, Kemp A, Sibert JR (1994). Patterns of scald injuries. Archives of Disease in Childhood.

[B5] Tennant WG, Davison PM (1991). Bath scalds in children in the south-east of Scotland. Journal of the Royal College of Surgeons of Edinburgh.

[B6] Feldman KW (1983). Help Needed on Hot Water Burns. Pediatrics.

[B7] Child Accident Prevention Trust (2002). Scarred for life.  Preventing bath water scalds in the home.  Discussion paper.

[B8] Huyer DW, Corkum SH (1997). Reducing the incidence of tap-water scalds: Strategies for physicians. Canadian Medical Association Journal.

[B9] Kemp A, Sibert J (1995). Preventing scalds to children. British Medical Journal.

[B10] Stephen FR, Murray JP (1991). The prevention of hot tap water burns--a study of electric immersion heater safety. Burns.

[B11] Georgieff K, Maw C (2004). The Wakefield District Burns and Scalds Prevention Project.

[B12] Katcher ML, Landry GL, Shapiro MM (1989). Liquid-crystal thermometer use in pediatric office counseling about tap water burn prevention. Pediatrics.

[B13] Katcher ML (1987). Prevention of tap water scald burns: evaluation of a multi-media injury control program. American Journal of Public Health.

[B14] Webne S, Kaplan BJ, Shaw M (1989). Pediatric burn prevention: an evaluation of the efficacy of a strategy to reduce tap water temperature in a population at risk for scalds. Journal of Developmental and Behavioral Pediatrics.

[B15] Waller AE, Clarke JA, Langley JD (1993). An evaluation of a program to reduce home hot tap water temperatures. Australian Journal of Public Health.

[B16] Erdmann TC, Feldman KW, Rivara FP, Heimbach DM, Wall HA (1991). Tap water burn prevention: the effect of legislation. Pediatrics.

[B17] Fallat ME, Rengers SJ (1993). The effect of education and safety devices on scald burn prevention. Journal of Trauma: Injury Infection and Critical Care.

[B18] Cagle KM, Davis JW, Dominic W, Ebright S, Gonzales W (2006). Developing a focused scald-prevention program. Journal of Burn Care & Research.

[B19] Spallek M, Nixon J, Bain C, Purdie DM, Spinks A, Scott D, McClure RJ (2007). Scald prevention campaigns: do they work?. Journal of Burn Care & Research.

[B20] Housing Corporation (2003). Scheme Development Standards.

[B21] Scottish Building Standards Agency Scottish Buildings Standards Agency Technical Handbook: Domestic. http://www.sbsa.gov.uk/tech_handbooks/th_html_2006/bsthd-79.htm.

[B22] Edwards P, Roberts I, Clarke M, DiGuiseppi C, Pratap S, Wentz R, Kwan I, R. C (2007). Methods to increase response rates to postal questionnaires. Cochrane Database of Systematic Reviews.

[B23] Anonymous (2003). STATA for Windows.

[B24] Ritchie J, Spencer L, A. Bryman, Burgess RG (1994). Qualitative data analysis for applied policy research. Analysing Qualitative Data.

[B25] Scottish Executive Adult literacy and numeracy in Scotland.. http://www.scotland.gov.uk/Publications/2001/07/9471/File-1.

[B26] Katz I, La Placa V, Hunter S Barriers to inclusion and succesful engagement of parents in mainstream services. http://www.jrf.org.uk/bookshop/ebooks/barriers-inclusion-parents.pdf.

[B27] Bailey N, Livingston M Population turnover and area deprivation.. http://www.jrf.org.uk/bookshop/eBooks/2004-population-census-deprivation.pdf.

